# Medicinal plants and traditional healing practices in ehotile people, around the aby lagoon (eastern littoral of Côte d'Ivoire)

**DOI:** 10.1186/s13002-015-0004-8

**Published:** 2015-03-14

**Authors:** Djah F Malan, Danho F R Neuba, Kouakou L Kouakou

**Affiliations:** UFR des Sciences de la Nature, Université Nangui Abrogoua, POB: 02 BP 801, Abidjan, 02 République de Côte d’Ivoire; Institut Botanique Aké-Assi d’Andokoi, POB: 08 BP 172, Abidjan, 08 Côte d’Ivoire

**Keywords:** Medicinal plants, Traditional knowledge, Ehotile, Côte d’Ivoire

## Abstract

**Background:**

Access to useful plants is a growing problem in Africa, increased by the loss of natural vegetation and the erosion of traditional knowledge. Ethnobotany contributes to promote these indigenous knowledge. Despite the large diversity of ethnic groups in Côte d’Ivoire, few ethnomedicine researches have targeted these groups. Among the great Akan group, the Ehotile people are one of the smallest and oldest ethnic group around the Aby Lagoon. The goal of this study was to analyze the level of knowledge and use of medicinal plants by the Ehotile people, and moreover, contribute to build a database about useful plants of first Ivorian people.

**Methods:**

Two sets of surveys were conducted in four Ehotile villages: a house-to-house freelist interview and an individual walk-in-the woods interview with some key informants identified by the community. Frequency of citation, Smith’s index, Use value and Informant Consensus Factor were used to estimate the local knowledge of medicinal plants.

**Results:**

Medicinal plants are widely used by Ehotile people. Some were used in addition to modern prescriptions while for some disorders commonly called “African diseases” only plants are used. 123 species employed in the treatment of 57 diseases were listed. Specifically, the most common indications included malaria, sexual asthenia, troubles linked to pregnancy, dysmenorrhoea and haemorrhoids. Analysis of freelists suggested that Ehotile people has a good knowledge of medicinal plants and the most salient included *Harungana madagascariensis*, *Alstonia boonei*, *Ocimum gratissimum* and *Xylopia acutiflora*. Regarding the consensus among key informants, ICF values were low (<0.5), however category of infectious and parasitic diseases obtained the best agreement (ICF = 0.42). Following the local experts, 4 types of plants availability were distinguished: Abundant plants easy to collect, abundant plants difficult to harvest, scarce plants and endangered plants.

**Conclusions:**

Despite the virtual disappearance of natural formations in Ehotile land, medicinal plants are important in the Ehotile health system. Medicinal plants are known and used alone or in addition to medical prescriptions to treat several ailments. However, some of them are becoming rare, and it is feared that this scarcity will result in the inevitable loss of associated knowledge and practices.

## Background

The contribution of traditional knowledge is invaluable in the long and costly process of developing drug molecules. Thanks to traditional medicine, more than 40% of commonly prescribed medicines throughout the world are directly or indirectly of natural origin [1]. The use of medicinal plants is still alive in African traditions. Indeed, medicinal plants serve as the primary source of healthcare for the majority of the Developing World’s population [2,3]. However, access to medicinal plants resources is a growing problem in Africa [3,4]. The loss of natural vegetation, due to various causes, is a major factor in the erosion of traditional knowledge.

Ethnobotany, field of Botany that analyzes the results of traditional uses of plants together with the cultural context in which the plants are used [5], contributes by its various approaches to preserve and promote this indigenous knowledge [6].

This approach that emphasizes on a user group (e.g. ethnic group) is not only still relevant [7], but also offers a greater opportunity to gather honest and reliable information in the field.

Many studies, in Africa that are dealing with medicinal uses of plants particularly in Côte d’Ivoire, are simple lists or concern ethnopharmacology. The main focus of studies into traditional pharmacopoeias is the perception of indigenous medicines and the ingredients used in their preparation or the chemical composition, pharmacology and toxicology of plants used [7].

The study of the use of plants, beyond the simple catalogue, using quantitative tools is extremely recent and works discussing the ultimate meaning of disease and the cultural dimension of its treatment are scarce in Côte d’Ivoire. In this country, the large diversity of ethnic groups contrasts with the paucity of ethnomedicine research targeting these groups. Yet, a few reference works [8-10] conducted among Ando, Bete, Abbey and Krobou people and major cross works [11,12] have shown that local specificities are also expressed through medicinal practices. In the complex ethnic mix that characterizes the bank of the Aby Lagoon (eastern littoral of Côte d’Ivoire), the Ehotile people (9 villages) differs from other Akan. The originality of these people is conferred by its territorial precedence, its history and lifestyle closely linked to the lagoon.

Some modern health facilities exist around the Aby lagoon. For example, the four large villages on the west bank of the Aby Lagoon (Etuessika, Melekoukro, N’galwa and Assomlan) have two rural health centres for an estimated 3,500 inhabitants. Each centre is served by one nurse and one midwife, i.e. one health worker for 875 inhabitants. This ratio is certainly significant compared to the Ivorian national ratio, which is 4.8 nurses and midwives per 10,000 inhabitants [13]. However, access to such centres is a major problem for some patients who have to travel a few kilometers or be evacuated by boat.

It’s known that when Western medicine is often absent or hard to access or too expensive, the use of remedies from plant becomes particularly significant [3,14]. In Ehotile area, this was a fact observed almost on a daily basis. Beside the State health workers, the traditional system of medicine is well established, based on healers whose reputation often goes beyond the borders of the village. For the four villages mentioned above, 15 confirmed healers were registered, i.e. one traditional health agent per 233 inhabitants [15]. The majority of these healers are *Komian* or acolytes of *Komian*. The *Komian* is a priest of traditional religion initiated according to the rites of an order which embodies invisible entities (*Boson*) whom he worships during public sessions of divination.

Moreover, the land occupied by Ehotile is one of the most degraded of the Ivorian Coast. Scarce natural vegetation that has withstood the plantations of coconut, oil palm or rubber is composed of the marshy patches and the islands of the Ehotile Islands National Park [16]. As the use of plants are often correlated with their availability [17], our study aimed to assess the knowledge and the use of medicinal plants (native or introduced regardless of their medicinal character) in the daily life by Ehotile people, contributing, therefore, to build an integrated database for expertise about useful plants of Ivorian people.

Beyond the study of the use and evaluation of the level of knowledge, our work also aims to probe the availability of the species used with local experts recognized in their community.

## Methods

### Study area

**Figure 1:**
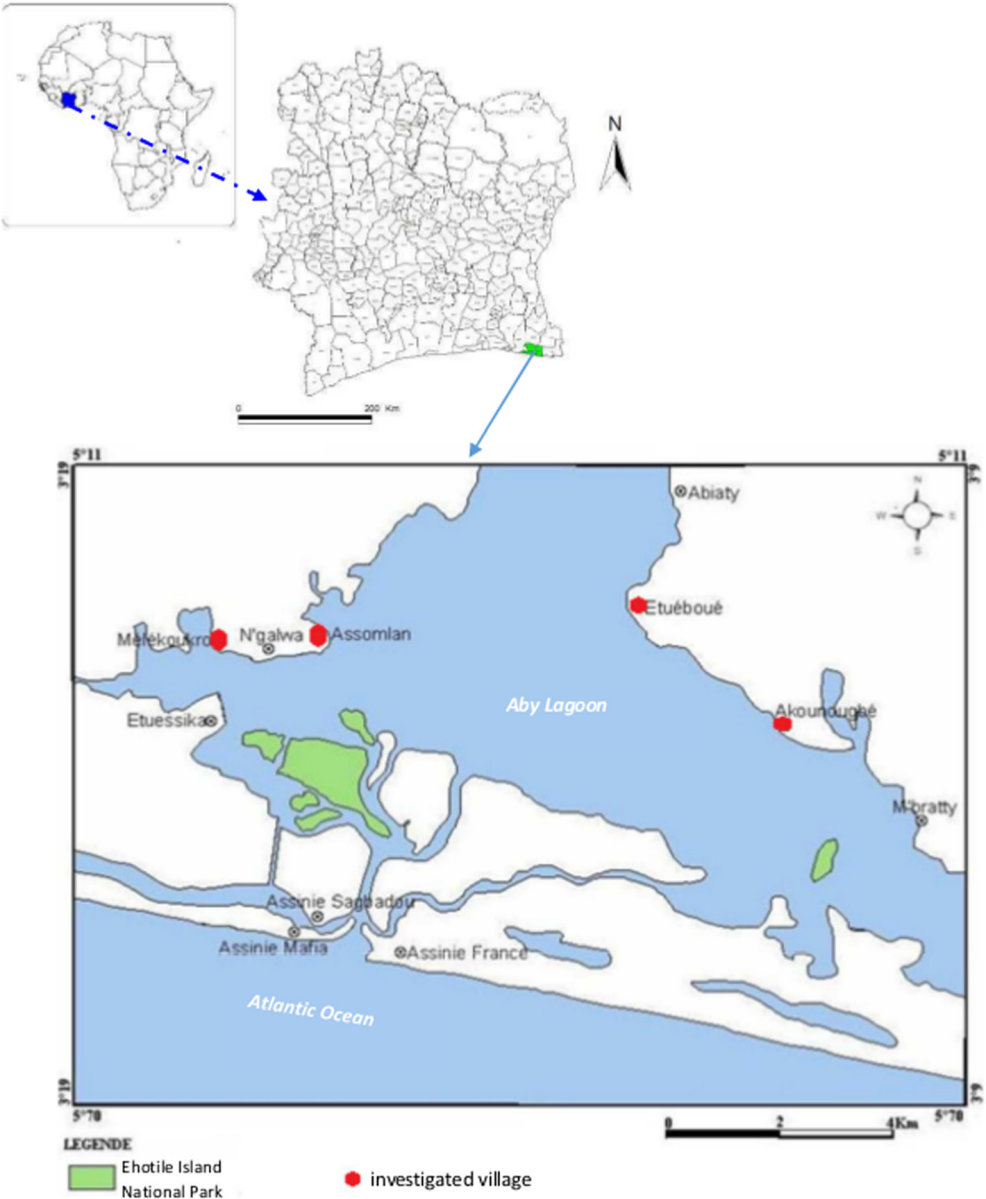
**Localisation of study area in Africa, Côte d’Ivoire and around the Aby lagoon.**

The Ehotile province is composed of 9 main villages round the Aby Lagoon. The survey was conducted in the four biggest, bordering the Ehotile Islands National Park: Mélékoukro, Assomlan (western bank) and Etuoboué and Akounougbé for the eastern bank (Figure 1). The climate of this zone is an equatorial transition type, characterized by four successive seasons: the long rainy season from March to July; the short dry season in August; the small rainy season from September to November and the large less rainy season, from December to February. The average annual rainfalls range from 1800 to 2000 mm and the annual average temperature is 26.4°C with a variation of 3°C.

The relief of the region is monotonous in the whole with some small plateaus of low altitudes (40 to 60 m) with irregular contours, separated by usually quite steep valleys. The soils in this area are generally hydromorphic on Quaternary or young marine sands with in some places, large accumulations of plant debris.

Concerning the vegetation, the study zone belongs to the Guinean Littoral Area [18]. The original vegetation (*Licania eleosperma* (Mildbr.) Prance & White and *Drypetes aframensis* Hutch. sub-littoral forest) has been replaced by cultivated areas. However, we can distinguish several types of vegetation including mangroves, coastal thickets, forests and plantations. In fact, in this area, due to the variety of soil conditions and trends, there is no dominant climax but a mosaic of edaphic groups.

Fishing is the main activity of the Ehotile people and until very recently (second quarter of the twentieth century), they were exclusively fishermen [19]. Fishing activity (individual or collective) is practiced by men and women. The men are responsible for fishing, the supply of firewood (mangrove wood, mostly) and women are engaged in fish preservation such as, smoking in readiness for marketing. Fishing activities are particularly intense during the long rainy season (May to July) and fall significantly in the dry season (January to April).

Agriculture is the second activity in the study area. From the stage of home consumption with food crops (cassava, yam, banana, etc.) it increased to the stage of cash crop with coconut, oil palm, pineapple and rubber. It is practiced by men and women. Women are mostly engaged in the cultivation of food (cassava and vegetables) while men are the owners of large plantations of coconut, oil palm, rubber or pineapple.

### Survey method

Two sets of surveys were carried out. The first survey was achieved during a house-to-house interview (N = 55, 30 women and 25 men, age ranging from 32 to 76 years, with an average age of 47.7). Questions were: What are the common diseases here? Do you know and use plants to cure these diseases? Do you know people specialized in traditional treatment of these diseases with plants? Through this step, an initial list of plants used was given and a list of 15 resource persons recognized for their competence in the treatment of various pathologies was established.

**Figure 2:**
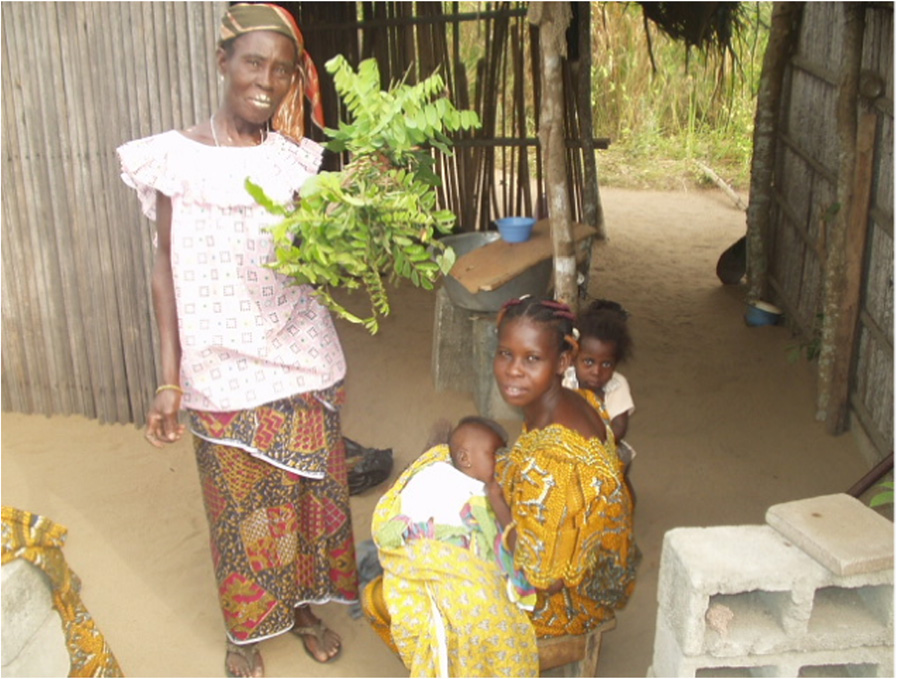
**Mrs. Mosoma, one of the ehotile key informants on medicinal plants, recognized as an expert of women infertility and diseases of newborn and infant.** She’s holding some plants, part of a recipe against infant fever attack.

The second survey involved some key informants selected among the previous list of identified resource persons (N = 8, 4 women and 4 men, age ranging from 46 to 70 years, with an average of 60 years). These informed persons were individually interviewed at home (Figure 2) or during a “Walk-in-the-woods” approach. This method involved walking with local people in the areas where they normally collected their medicinal plants while interviewing them [20]. Information on local names of plants and practices was collected. With these key informants who were often reluctant to give their medicinal recipes, our entry point was the availability of resources used. These resource persons followed the dynamics of the surrounding vegetation and knew the common species in their region, those which were rare and those which newly appeared. The collected vouchers were identified at the Herbarium of Ivorian National Floristic Centre (ABJ) and confirmed by the Late Prof. Aké Assi Laurent. Voucher specimens were deposited at the Laboratory of Botany of Nangui Abrogoua University (Abidjan) and at the Institut de Botanique Aké Assi d’Andokoi (Abidjan).

### Evaluation of local knowledge

There are several indices to scientifically estimate indigenous knowledge in a specific field [21]. However, the most commonly used are those based on informant consensus, i.e. the level of agreement among various interviewees (see [21-23]). These indices, using spontaneous quotations, are based on the principle that the greater the salience of a given plant or use in the community, the more likely it is to be mentioned [23,24].

One of the simplest and best known is the frequency of citation (Fq), i.e. the number of informants who mentioned a given species. It is a good index to evaluate the credibility of collected information and the level of knowledge within a surveyed population [25]. However, this index does not take into account the rank of appearance of an item in the list of citations. So the Smith’s index [25-27] was used. It is based on the frequency of citation, the rank and the length of citation lists and it ranges from 0 to 1 (maximum importance). The formula reviewed [27] is:

$$ S=\left\{{\displaystyle \sum \left[\left({L}_i-{R}_j+1\right)/{L}_j\right]}\right\}/N $$

where *S* is the salience of an individual item (Smith’s index), *Li* the length of each informant’s list, and *Rj* the rank of the item (here, the plant cited) in that list. In addition, the use-value (UV) was calculated for each plant using the following formula [22,28], modified from [29]:

$$ UV={\displaystyle \sum \frac{U_i}{N}} $$

where *U*_*i*_ is the number of uses mentioned by each informant for a given species and N is the total number of informants. Use-value as opposed to the relative importance (RI) greatly emphasizes species that have many uses, even if those uses are known only by a few people [22].

Frequency of citation, Smits’s Index and use-value were used for house-to-house data analysis. Furthermore, the Informant Consensus Factor (ICF) was calculated, as follow [29], to assess the agreements among key informants:

$$ ICF=\frac{n_{ur}-{n}_t}{n_{ur}-1} $$

where *n*_*ur*_ is number of use citation in each category and *n*_*t*_ number of species used. ICF ranges from 0 (plants are chosen randomly, or informants do not exchange information about their use) to 1 (there is a well-defined selection criterion in the community and/or if information is exchanged between informant [29,30]).

Diseases identified were classified into broad categories (considered here as categories of use). The classification was adapted from the 10^th^ version International Statistical Classification of Diseases and related Health Problems or ICD-10 [31].

### Ethical consideration

Before being interviewed, local residents of each investigated village were briefed on the research project during an agreement meeting and educated prior informed consent was established following the recommendations of the International Society of Ethnobiology Code of ethics for the publication of this research and any accompanying images. Meetings were held in the village chief's court and the conclusions were sealed, each time with a libation, following the Ehotile customary protocol.

## Results

### Observations on Ehotile medicinal practices

Generally, plants constitute a major part in the health system of Ehotile people. Information on popular medicinal recipes is easily accessible. Among the diseases listed in our work, some are subject to a complementary treatment (modern prescriptions and medicinal plants) including malaria, diarrhoea, and headache. The first treatment is usually provided by the housewives. That is why basic medicinal plants such as *Ocimum gratissimum* L., *Eclipta prostrata* (L.) L., *Capsicum frutescens* L. are grown in the homegardens or backyards in village settings. These plants constitute a kind of living “pharmacy box”.

Beside diseases for which complementarity between traditional and modern medicines was observed, there are some disorders commonly called “African-diseases” for which modern medicines are not sought. For these, it is the healer who is solicited. In some cases, the complexity of the described symptoms or their non-organic origin has led to the retention of the local names of these ailments: *Asema*, *elokposan* or *butumanklan*.

Finally, there is a last category of diseases where treatment offered in health centres is not accepted by villagers. These illnesses include haemorrhoids, hydrocephalus or disorders of reproductive health (sexual asthenia, impotence or infertility).

### Plants used in common traditional medicine by Ehotile people

The investigation yielded a list of 123 plant species, 96 after the house-to-house survey and 27 only collected with local experts (Table 1). These taxa were distributed in 113 genera and 52 plant families (44 of Dicotyledons, 6 of Monocotyledons and 2 of Pteridophytes). The richest ones were Leguminosae (15 species), Euphorbiaceae (9), Annonaceae (7), Apocynaceae (6) and Compositae (6).

**Table 1:** **List of medicinal plants used by Ehotile people**

**Species**	**Voucher number**	**Family**	**Informant**	**Fq**	**Smiths’ S**	**Indication**	**Part used and administration mode**
*Abrus precatorius* L.	Malan 758	Leguminosae	LP	0.04	0.015	Ophtalmia	Eye drops with leaf juice
						Palpitations	Leaf juice swallowed
*Adenia lobata* (Jacq.) Engl.	Malan 504	Passifloraceae	LP	0.04	0.032	Ophtalmia	Eye drops with sap
*Ageratum conyzoides* L.	AD 128	Compositae	LP	0.04	0.016	Headache	Nose drops with leaf juice
*Albertisia cordifolia* (Mangenot & Miège) Forman	Malan 638, 718	Menispermaceae	LP, KI	0.07	0.04	Oedema	Topical application with crushed leaves
*Albizia zygia* (DC.) J. F. Macbr.	Malan 596	Leguminosae	LP, KI	0.02	0.018	Rheumatism	Topical application with crushed leaves
*Alchornea cordifolia* (Schumach. & Thonn.) Müll.Arg.	AD 224	Euphorbiaceae	LP, KI	0.05	0.047	White discharge	Enema with leaves mashed and diluted in water
						Malaria	Leaves decoction drunk and taken as enema
						Wound	Topical application with crushed leaves
						Sexual asthenia	Leaves decoction drunk
*Alstonia boonei* (DC.) Willd.	Malan 630	Apocynaceae	LP, KI	0.35	0.294	Malaria	Leaves and bark decoction drunk and taken as enema
*Alternanthera pungens* Kunth	AD 132	Amaranthaceae	LP, KI	0.05	0.038	Convulsion	Body smeared with leaves mashed
						Infant diarrhoea	Enema with leaves mashed and diluted in water
*Anchomanes difformis* (Blume) Engl.	Malan 570	Araceae	LP	0.04	0.023	Dysmenorrhoea	Enema with tuber mashed and diluted in water
						Pregnancy care	
*Anthocleista nobilis* G.Don	AD 63	Gentianaceae	LP, KI	0.04	0.034	Malaria	Body smeared with mashed bark
						Rheumatism	Topical application with crushed roots
*Antiaris toxicaria* (Engl.) C.C. Berg	Malan 600	Moraceae	LP, KI	0.04	0.016	Hydrocephalus	Bathing with bark decoction
*Azadirachta indica* A.Juss.		Meliaceae	LP, KI	0.07	0.053	Malaria	Body smeared with mashed leaves, leaves decoction drunk
*Bambusa vulgaris* Wendel. ex Nees	Malan 447	Poaceae	LP, KI	0.09	0.06	Malaria	Leaves decoction drunk
*Baphia nitida* Lodd.	AD 166	Leguminosae	LP, KI	0.11	0.08	Bubo	Topical application with crushed leaves
						Bad breath	Roots as chewing stick
						Boil	Topical application with crushed leaves
						Lumbar pain	Enema with mashed roots diluted in water
*Bidens pilosa* L.	AD 112	Compositae	LP, KI	0.09	0.061	Hydrocephalus	Body smeared with mashed leaves Enema with leaves mashed and diluted in water
						Diarrhoea	
						Malaria	
						Giddiness	
*Blighia sapida* Koenig	AD 196	Sapindaceae	LP	0.04	0.026	Lumbar pain	Enema with bark mashed and diluted in water
						Malaria	
						Rheumatism	Topical application with crushed bark
*Blighia welwitschii* (Hiern) Radlk.	Malan 968	Sapindaceae	KI	-	-	Malaria	Enema with bark mashed and diluted in water Topical application with crushed bark
						Rheumatism	
						Oedema	
*Bridelia micrantha* (Hochst.) Baill.	AD 89	Phyllanthaceae	LP	0.04	0.031	Malaria	Enema with leaves mashed and diluted in water
*Caesalpinia bonduc* (L.) Roxb.	AD 240	Leguminosae	LP, KI	0.05	0.045	Malaria	Enema with leaves mashed and diluted in water
						Sexual asthenia	
*Cajanus cajan* (L.) Roxb.	AD 13	Leguminosae	LP, KI	0.02	0.012	Malaria	Bathing with leaves decoction
*Capsicum frutescens* L.		Solanaceae	KI	0.04	0.018	Serious disease of unknown origin	Ocular instillations with leaf juice
						Contraception	Enema with fruits mashed and diluted in water
*Carica papaya* L.		Caricaceae	LP, KI	0.05	0.028	Contraception	Enema with leaves mashed and diluted in water
						Malaria	Leaves decoction drunk
*Carpolobia lutea* G.Don	AD07	Polygalaceae	LP, KI	0.05	0.037	Sexual asthenia	Roots decoction drunk
*Cassia alata* L.	AD 15	Leguminosae	LP, KI	0.18	0.1	Dry patch	Topical application with crushed leaves
						Malaria	Body smeared with leaves mashed
						Constipation	Leaves decoction drunk
*Cassia occidentalis* L.	Malan 618	Leguminosae	LP, KI	0.07	0.04	Headache	Nose drops with leaf juice
						Malaria	Leaves decoction drunk
*Catharanthus roseus* (L.) G.Don		Apocynaceae	LP	0.02	0.015	Inguinal hernia	Enema with mashed roots diluted in water
*Ceiba pentandra* (L.) Gaertn.	Malan 1001	Malvaceae	LP, KI	0.02	0.009	Diarrhoea	Enema with mashed leaves or bark diluted in water
*Chassalia koly* (Schumach.) Hepper	Malan 717	Rubiaceae	LP, KI	0.02	0.011	Headache	Eye drops with juice from roots
*Cleistopholis patens* (Benth.) Engl. & Diels	AD 68	Annonaceae	LP, KI	0.11	0.07	Rheumatism *Elokposan*	Topical application with crushed bark
*Cnestis ferruginea* Vahl ex DC.	Malan 395	Connaraceae	LP, KI	0.05	0.037	Hydrocephalus	Bathing with leaves decoction
						Pregnancy care	Enema with roots mashed and diluted in water
*Cocos nucifera* L.		Arecaceae	LP	0.02	0.017	Raging toothache	Mouthwash with roots decoction
						*Butumanklan*	Enema with roots mashed and diluted in water
*Cola lateritia var. maclaudi* (A.Chev.) Brenan & Keay	AD 176, Malan 812	Malvaceae	LP	0.02	0.014	Malaria	Body smeared with mashed leaves
*Cola nitida* (Vent.) Schott & Endl.	AD 163	Malvaceae	LP	0.02	0.009	Whitlow Chickenpox	Topical application with crushed bark
*Costus afer* Ker-Gawl.	Malan 1118	Costaceae	LP, KI	0.02	0.008	Palpitations	Oral instillations with crushed leaves
*Dacryodes klaineana* (Pierre) Lam.	Malan 777, 898	Burseraceae	LP, KI	0.02	0.007	Dysmenorrhoea	Enema with bark mashed and diluted in water
*Daniellia thurifera* Benn.	Malan 697	Leguminosae	LP, KI	0.05	0.026	Post-partum care	Bathing with leaves decoction
*Desmodium adscendens* (Sw.) DC.	Malan 443	Leguminosae	LP, KI	0.05	0.04	Pregnancy care	Enema with leaves mashed and diluted in water
						Diarrhoea	
						Wound	Topical application with crushed leaves
*Digitaria horizontalis* Willd.	Malan 874	Poaceae	LP, KI	0.05	0.04	Oedema	Body smeared with mashed whole plant
*Dioclea reflexa* Hook.f.	Malan 994	Leguminosae	LP, KI	0.02	0.009	Infant fortification	Entire seed soaked in infant bathing water
*Diospyros sanza-minika* A.Chev.	Malan 809	Ebenaceae	LP, KI	0.02	0.003	Giddiness	Topical application with crushed bark
*Eclipta prostrata* (L.) L.	Malan 752	Compositae	LP, KI	0.07	0.041	Hydrocephalus	Body smeared with leaves mashed
						*Asema*	Bathing with leaves decoction
						Giddiness	Eye drops with leaf juice
						Pregnancy care	Enema with leaves mashed and diluted in water
						Malaria	
						Diarrhoea	
*Elaeis guineensis* Jacq.		Arecaceae	LP, KI	0.02	0.01	Abortion	Enema with mashed roots diluted in water
*Erythrina senegalensis* DC.	Malan 856	Leguminosae	LP	0.02	0.006	Rheumatism	Topical application with crushed bark
						Oedema	Body smeared with mashed leaves
						Dysmenorrhoea	Enema with leaves mashed and diluted in water
*Euadenia trifoliolata* (Schumach. & Thonn.) Oliv.	Malan 695	Capparaceae	LP, KI	0.02	0.009	Haemorrhoids	Enema with mashed roots diluted in water
*Eugenia whytei* Sprague	Malan 897	Myrtaceae	LP, KI	0.05	0.021	Bad breath	Roots as chewing stick
						Infant diarrhoea	Enema with mashed leaves diluted in water
						Nausea	Leaves chewed
*Euphorbia hirta* L.	AD 25	Euphorbiaceae	LP, KI	0.11	0.048	Measles	Body smeared with mashed leaves
						Pregnancy care	Enema with whole plant mashed and diluted in water
						Diarrhoea	
*Ficus exasperata* Vahl	AD 48	Moraceae	LP	0.02	0.009	Injury	Topical application with crushed roots
*Flagellaria guineensis* Schumach.	AD 04	Flagellariaceae	LP, KI	0.04	0.005	Malaria	Bathing with leaves decoction
						Delay of motor function	Enema with roots mashed and diluted in water
*Garcinia kola* Heckel	AD 45	Clusiaceae	LP	0.04	0.025	Bad breath	Roots as chewing stick
						Sexual asthenia	
*Glyphaea brevis* (Spreng.) Monachino	AD 206	Malvaceae	LP	0.02	0.011	Pregnancy care	Enema with leaves mashed and diluted in water
*Gossypium barbadense* L.	AD 177	Malvaceae	LP, KI	0.02	0.008	Dysmenorrhoea	Enema with leaves mashed and diluted in water
*Hallea ledermannii* (K.Krause) Verdc.	Malan 787, AD 227	Rubiaceae	LP, KI	0.04	0.013	Malaria	Bathing with bark decoction
						Convulsion	Body smeared with mashed bark
*Harungana madagascariensis* Lam. ex Poir.	Malan 719	Hypericaceae	LP, KI	0.53	0.38	Malaria	Bark decoction drunk, taken as enema and for bathing, Body smeared with mashed bark
*Heterotis rotundifolia* (Sm.) Jacq.-Fél.	Malan 552	Melastomataceae	LP, KI	0.05	0.04	Diarrhoea	Enema with leaves mashed and diluted in water
*Hoslundia opposita* Vahl	Malan 953	Lamiaceae	LP	0.02	0.016	Convulsion	Eye drops with leaf juice
*Icacina mannii* Oliv.	Malan 1042	Icacinaceae	LP, KI	0.15	0.083	Dysmenorrhoea	Tuber decoction drunk
						Sexual asthenia	
						Haemorrhoids	
*Imperata cylindrica* (L.) Raeuschel	Malan 458	Poaceae	KI	-	-	Convulsion	Body smeared with mashed leaves
*Jatropha curcas* L.	AD 58	Euphorbiaceae	LP	0.02	0.009	Abortion	Enema with leaves mashed and diluted in water
*Jatropha gossypiifolia* L.	AD 139	Euphorbiaceae	LP	0.02	0.004	Dysmenorrhoea	Enema with leaves mashed and diluted in water
*Khaya ivorensis* A.Chev.	Malan 419	Meliaceae	LP	0.04	0.019	Haemorrhoids	Enema with bark mashed and diluted in water
						Sexual asthenia	Bark soaked in local alcohol and drunk
*Kigelia africana* (Lam.) Benth.	AD 201	Bignoniaceae	LP, KI	0.07	0.048	Swollen breasts	Topical application with fruits, leaves and bark crushed
						Sterility	Enema with bark mashed and diluted in water
*Lannea nigritana* (Scott-Elliot) Keay	AD 95	Anacardiaceae	LP	0.02	0.006	Ophtalmia	Eye drops with leaf juice
						Toohtache	Mouthwash with leaves decoction
*Lophira alata* Banks ex Gaertn. f.	Malan 340	Ochnaceae	LP	0.04	0.012	Bad breath	Roots as chewing stick
						*Elokposan*	Bathing with bark decoction
						Malaria	
*Mallotus oppositifolius* (Geisel.) Müll.Arg.	AD 278	Euphorbiaceae	LP	0.02	0.007	*Asema*	Bathing with leaves decoction
*Mangifera indica* L.		Anacardiaceae	KI	-	-	Malaria	Bathing with bark decoction
*Manihot esculenta* Crantz		Euphorbiaceae	LP, KI	0.09	0.022	Headache	Nose drops with leaf juice
						Ophtalmia	Eye drops with leaf juice
						Infant diarrhoea	Enema with roasted fruits mashed and diluted in water
						Diarrhoea	
						Gonorrhoea	Enema with leaves mashed and diluted in water
*Manotes longiflora* Baker	Malan 886	Connaraceae	LP	0.04	0.006	Ophtalmia	Eye drops with leaf juice
						Dysenteria	Enema with leaves mashed and diluted in water
*Mareya micrantha* (Benth.) Müll.Arg.	Malan 734	Euphorbiaceae	KI	-	-	Childbirth	Leaves decoction drunk
*Microdesmis keayana* Léonard	Malan 659	Pandaceae	LP, KI	0.05	0.014	Bad breath	Roots as chewing stick
						Dysmenorrhoea	Enema with leaves mashed and diluted in water
*Microglossa pyrifolia* (Lam.) O.Ktze	AD 92	Compositae	LP, KI	0.07	0.044	Hydrocephalus	Bathing with leaves decoction, body smeared with mashed leaves, leaves decoction drunk
						*Asema*	Bathing with leaves decoction
						Convulsion	Nose drops with leaf juice, body smeared with mashed leaves
						Pregnancy care	Enema with leaves mashed and diluted in water
						Dysmenorrhoea	
*Microgramma owariensis* (Desv.) Alston	AD 39	Polypodiaceae	LP, KI	0.05	0.028	Sterility	Enema with whole plant mashed and diluted in water
						Dysmenorrhoea	
*Microsorum scolopendria* (Burm.f.) Copel.	AD 57	Polypodiaceae	LP, KI	0.04	0.014	Dysmenorrhoea	Enema with whole plant mashed and diluted in water
*Milicia excelsa* (Welw.) C.C.Berg	Malan 785	Moraceae	KI	-	-	Hydrocephalus	Bathing with bark decoction, body smeared with mashed bark
						Dysenteria	Enema with mashed bark diluted in water
						*Butumanklan*	
*Mitracarpus scaber* Zucc.	AD 156	Rubiaceae	LP	0.01	0.018	Dry patchs	Topical application with crushed leaves
						Lumbar pain	Enema with mashed leaves diluted in water
*Momordica charantia* L.	Malan 1011	Cucurbitaceae	LP, KI	0.11	0.029	Malaria	Bathing with leaves decoction, body smeared with mashed leaves
						Chickenpox	
						Abortion	Body smeared with mashed leaves, Enema with seeds mashed and diluted in water
*Monodora myristica* (Gaertn.) Dunal	Malan 780	Annonaceae	LP, KI	0.01	0.029	Haemorrhoids	Seeds added to enema preparation
*Morinda lucida* G.Don	AD 175	Rubiaceae	KI	-	-	Infant diarrhoea	Enema with bark mashed and diluted in water
*Musa paradisiaca* L.		Musaceae	KI	-	-	Malaria	Bathing with leaves decoction
						Ophtalmia	Eye drops with sap
*Nephrolepis biserrata* (Sw.) Schott	Malan 599	Nephrolepidaceae	LP, KI	0.05	0.009	Remove splinters	Topical application with crushed leaves
						Dysmenorrhoea	Enema with leaves mashed and diluted in water
*Newbouldia laevis* (P. Beauv.) Seem. ex Bureau	Malan 1027	Bignoniaceae	LP, KI	0.05	0.024	Dysmenorrhoea	Enema with leaves mashed and diluted in water
						Hoarseness	Bark decoction drunk
						Raging toothache	Mouthwash with bark decoction
						Dysmenorrhoea	Enema with mashed bark diluted in water
						Remove splinters	Topical application with crushed leaves
						Fracture	Topical application with crushed bark
*Nymphaea lotus* L.	Malan 925	Nympheaceae	LP	0.04	0.008	Pregnancy care	Enema with leaves mashed and diluted in water
						Delay of motor function	
*Ocimum americanum var. americanum* L.	AD 16	Lamiaceae	LP, KI	0.04	0.014	Lumbar pain	Enema with leaves mashed and diluted in water
						Swollen breasts	Topical application with crushed leaves
						Convulsion	Eye drops with leaf juice
*Ocimum gratissimum* L.	Malan 1009	Lamiaceae	LP, KI	0.38	0.245	Convulsion	Eye drops with leaf juice
						Headache	Nose drops with leaf juice
						Cough	Nose or eye drops with leaf juice
						Fever attack	Enema with mashed leaves diluted in water
						Malaria	
						Dysmenorrhoea	
						Pregnancy care	
						Infant diarrhoea	
						Stomachache	
*Palisota hirsuta* (Thunb.) Engl.	Malan 984	Commelinaceae	KI	-	-	Delay of motor function	Enema with leaves mashed and diluted in water
						Dysmenorrhoea	
*Parinari excelsa* Sabine	AD 148	Chrysobalanaceae	KI	-	-	Sexual asthenia	Bark soaked in local alcohol and drunk
						Haemorrhoids	Enema with bark mashed and diluted in water
						Dysmenorrhoea	
*Paullinia pinnata* L.	AD 124, Malan 853	Sapindaceae	LP, KI	0.2	0.122	Hydrocephalus	Bathing with leaves decoction
*Pentaclethra macrophylla* Benth.	Malan 1110	Leguminosae	LP, KI	0.05	0.03	Hydrocephalus	Bathing with bark decoction, body smeared with mashed bark
						Malaria	Body smeared with mashed bark
*Phaulopsis ciliata* (Willd.) Hepper	Malan 954	Acanthaceae	KI	-	-	Colic	Enema with leaves mashed and diluted in water
*Phyllanthus amarus* Schumach. & Thonn.	AD 144	Phyllanthaceae	LP, KI	0.13	0.037	Chickenpox	Body smeared with whole plant mashed
						Pregnancy care	Enema with leaves mashed and diluted in water
						Malaria	Whole plant decoction drunk
*Picralima nitida* (Stapf) T. & H.Durand	AD 237	Apocynaceae	LP, KI	0.09	0.082	Inguinal hernia	Enema with seeds mashed and diluted in water
*Piptadeniastrum africanum* (Hook.) Brenan	Malan 1108	Leguminosae	KI	-	-	Inguinal hernia	Enema with bark mashed and diluted in water
*Pleioceras barteri* Baill.	Malan 700	Apocynaceae	KI	-	-	Boil	Topical application
						Oedema	
*Plumbago zeylanica* L.	Malan 873	Plumbaginaceae	LP, KI	0.04	0.004	Inguinal hernia	Enema with leaves and roots mashed and diluted in water
*Psidium guajava* L.		Myrtaceae	KI	-	-	*Butumanklan*	Enema with roots mashed and diluted water
*Pterocarpus santalinoides DC.*	AD 09, AD 59	Leguminosae	KI	-	-	Malaria	Body smeared with mashed bark
*Pycnanthus angolensis* (Welw.) Warb.	AD 212	Myristicaceae	LP, KI	0.05	0.022	Haemorrhoids	Enema with bark mashed and diluted in water
						Anaemia	Bark decoction drunk
*Rauvolfia vomitoria* Afzel.	AD 36	Apocynaceae	LP, KI	0.07	0.018	Malaria	Body smeared with mashed roots, enema with roots mashed and diluted in water
						Sexual asthenia	Roots soaked in local alcohol and drunk
						Inguinal hernia	Enema with mashed roots diluted in water
						Rheumatism	Topical application with crushed bark
*Rhizophora racemosa* G.F.W.Mey.	Malan 806	Rhizophoraceae	KI	-	-	Hydrocephalus	Bathing with bark decoction and body smeared with mashed bark
*Ricinodendron heudelotii* (Baill.) Pierre ex Heckel	Malan 669	Euphorbiaceae	LP	0.04	0.004	Dysmenorrhoea	Enema with bark mashed and diluted in water
						Haemorrhoids	
*Ricinus communis* L.		Euphorbiaceae	KI	-	-	Headache	Topical application with mashed bark
						Lumbar pain	Massage with warm leaves
*Saccharum officinarum* L.		Poaceae	KI	-	-	Abortion	Enema with leaves mashed and diluted in water
*Secamone afzelii* (Schultes) K.Schum.	AD 375	Apocynaceae	KI	-	-	Lumbar pain	Enema with leaves mashed and diluted in water
						Asthma	Leaves decoction drunk
*Smeathmannia pubescens* Soland. ex R.Br.	AD 171, Malan 863	Passifloraceae	KI	-	-	Bad breath	Roots as chewing stick
*Solanum lycopersicum* L.		Solanaceae	LP, KI	0.07	0.018	Serious disease of unknown origin	Eye drops with leaf juice
						Injury	Topical application with crushed leaves
						Contraception	Enema with leaves mashed and diluted in water
*Solanum nigrum* L.	Malan 1022	Solanaceae	LP	0.04	0.002	Malaria	Enema with leaves mashed and diluted in water
*Solenostemon monostachyus* (P.Beauv.) Briq	Malan 982	Lamiaceae	KI	-	-	Laryngitis	Leaf juice swallowed
						Haedache	Nose drops with leaf juice
						Mental illness	
*Spathodea campanulata* P.Beauv.	AD 116	Bignoniaceae	LP	0.05	0.035	Malaria	Bark decoction drunk, taken as enema and for bathing, body smeared with mashed bark
*Spondias mombin* L.	AD 217	Anacardiaceae	LP	0.04	0.017	Diarrhoea	Enema with leaves mashed and diluted in water
						Post-partum care	
*Strombosia pustulata var. lucida* (Léonard) Villiers	Malan 816	Olacaceae	KI	-	-	Bad breath	Roots as chewing stick
*Syzygium guineense var. littorale* Keay	AD 140, AD 158	Myrtaceae	LP	0.05	0.011	*Butumanklan*	Enema with bark mashed and diluted in water
						Diarrhoea	
						Stomachache	
*Tetracera alnifolia* Willd.	AD 232	Dilleniaceae	KI	-	-	Inner ear cleanning	Ear drops with sap
*Tetrapleura tetraptera* (Schumach. & Thonn.) Taub.	AD 143	Leguminosae	KI	-	-	Impotence	Enema with bark mashed and diluted in water
*Tiliacora dinklagei* Engl.	Malan 884	Menispermaceae	KI	-	-	Sexual asthenia	Roots soaked in local alcohol and drunk
*Trema guineensis* Schumach. & Thonn.	Malan 676	Cannabaceae	LP	0.04	0.005	Stomachache	Enema with leaves mashed and diluted in water
						Pregnancy care	
*Uvaria afzelii* Scott-Elliot	Malan 793, AD 62	Annonaceae	LP, KI	0.22	0.123	Hydrocephalus	Bathing with decoction of roots and leaves, body smeared with leaves and roots mashed
						Pregnancy care	Enema with leaves mashed and diluted in water
						Colic	Decoction of leaves drunk
*Uvaria chamae* P.Beauv.	Malan 703, AD 106	Annonaceae	LP, KI	0.09	0.068	Diaper dermatitis	Body smeared with roots mashed
						Measles	Topical application with crushed roots and leaves
						Chickenpox	Body smeared with mashed roots
*Uvariastrum insculptum* (Engl. & Diels) Spargue & Hutch.	Malan 912	Annonaceae	KI	-	-	Bad breath	Roots as chewing stick
						Sexual asthenia	Roots soaked in local alcohol and drunk
*Vernonia amygdalina* Delile	AD 131	Compositae	LP, KI	0.11	0.051	Diarrhoea	Enema with leaves mashed and diluted in water
						Helminthiasis	
						Malaria	Bathing with leaves decoction, body smeared with mashed leaves
						Chickenpox	
*Vernonia conferta* Benth.	AD 187	Compositae	LP, KI	0.07	0.059	Malaria	Leaves decoction drunk
*Vitex oxycuspis* Baker	AD 234	Lamiaceae	KI	-	-	Haemorrhoids	Enema with bark mashed and diluted in water
*Xylopia acutiflora* (Dunal) A.Rich.	Malan 916	Annonaceae	LP	0.04	0.016	Bad breath	Roots as chewing stick
						Sexual asthenia	Roots soaked in local alcohol and drunk
*Xylopia aethiopica* (Dunal) A.Rich.	Malan 711	Annonaceae	LP, KI	0.29	0.225	Post-partum care	Enema with fruits mashed and diluted in water
*Zanthoxylum gilletii* (De Wild.) Waterman	Malan 742	Rutaceae	LP, KI	0	0.111	Fever attack	Body smeared with mashed bark
						Sexual asthenia	Bark soaked in local alcohol and drunk
						Laryngitis	Bark decoction drunk
						Malaria	Body smeared with mashed bark
						Cough	Bark decoction drunk
						Colic	Enema with bark mashed and diluted in water
						Haemorrhoids	
						Post-partum care	

Frequency of citation (Fq) and Smith’ index (Smith’ S), only for the survey with laypeople (LP). KI: key informant.

All morphological types are represented but with a high prevalence of trees and shrubs (61.8%) followed by lianas (22.8%) and herbaceous species (15.4%). Most of the plants used were native species (85.3%) though few exotic species were recorded (14.7%). These exotic plants included mango tree (*Mangifera indica* L.), papaya (*Carica papaya* L.), guava (*Psidium guajava* L.), plantain (*Musa paradisiaca* L.), introduced for their edible fruit and neem tree (*Azadirachta indica*), introduced as ornamental or shade plant. These plants, over time, play an important role in traditional African medicines.

**Figure 3:**
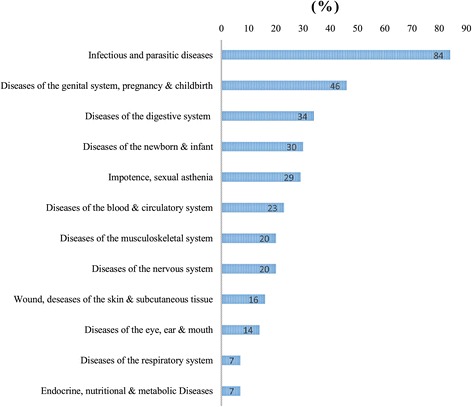
**Diseases categories in relation to medicinal plants used by ehotile people.**

All the plants listed were used for 59 indications (9 exclusively from key informants, 11 from laypeople and 39 from both groups) sorted in 12 categories (Figure 3). The most cited category is the group of infectious and parasitic diseases (84%), followed remotely by the diseases of the genital system, pregnancy and childbirth (46%) and the diseases of the digestive system (34%). Categories of endocrine, nutritional & metabolic diseases and diseases of the respiratory system are the least common with only 7% of the citations.

Specifically, the most common indications included malaria (82% of citations), sexual weakness (29%), development of pregnancy (23%), dysmenorrhoea (22%) and haemorrhoids (21%).

Analysis of free lists suggested that Ehotile people, in general, has a good knowledge of medicinal plants. The length of the lists ranged from 1 (only one informant) to 38 plants, with an average of 7.1 plants per list. However, frequent lengths were 3 plants per list (25.5% of informants) and 4 per list (18.2% of informants).

Regarding the salience of plants utilized, *Harungana madagascariensis* Lam. ex Poir. showed the highest Smith’s index (S = 0.38). This plant was widely cited (Fq = 0.53) but with an average rank of 3.9 and a low use-value (UV = 0.02). *H. madagascariensis* was followed by *Alstonia boonei* De Wild. (S = 0.32; Fq = 0.35, average rank = 1.8 and UV = 0.02), *Ocimum gratissimum* L. (S = 0.24; Fq = 0.38, average rank = 3.57 and UV = 0.13) and *Xylopia acutiflora* (Dunal) A.Rich. (S = 0.22; Fq = 0.29, average rank = 3.88 and UV = 0.04).

Seven types of drugs were listed in very different proportions including leaves (42%), barks (25%), roots and tubers (19%), fruits and seeds (8%), whole plant (4%), inflorescences (1%) and exudates (1%). Anal route using enema bag (33.2%), oral route (27.4%) and body brushing (13.9%) constituted the main administration routes.

### Key informant consensus and resources availability

The survey among specialists permitted to collect 92 species of which 27 are specific to this group, for 48 therapeutic indications. These specific plants included *Antiaris toxicaria* Lesch. *Blighia welwitschii* (Hiern) Radlk., *Chassalia kolly* (Schumach.) Hepper, *Costus afer* Ker Gawl., *Diospyros sanza*-*minika* A.Chev., *Euadenia trifoliolata* (Schumach. & Thonn.) Oliv., *Mareya micrantha* (Benth.) Müll. Arg., *Microgramma owariensis* (Desv.) Alston, *Vernonia conferta* Benth., *Plumbago zeylanica* L., *Ocimum americanum* L. (Fq = 1), *Icacina mannii* Oliv. (Fq = 0.75), *Uvaria afzelii* G.F. Scott-Elliot (Fq = 0.75), *Zanthoxylum gilletii* (De Wild.) P.G.Waterman (Fq = 0.63) and *Microdesmis keayana* J.Léonard (Fq = 0.63).

ICF ranges from 0 to 0.42, implying that there is no real consensus in the medicinal use of plants (Table 2). However, the highest values were recorded for infectious & parasitic diseases (ICF = 0.42), diseases of the blood & circulatory system (ICF = 0.30). The lowest ICF were recorded for diseases of the eye, ear (ICF = 0) & mouth and diseases of the respiratory system (ICF = 0). In the mental and behavioural disorders, only one indication was given for a single plant: *Solenostemon monostachyus* (P.Beauv.) Briq., used to treat or calm people with mental disorders.

**Table 2:** **Key informant consensus by diseases category**

**Category**	**Species**	**Citation**	**ICF**
Infectious & parasitic diseases	31	53	0.42
Diseases of the blood & circulatory system	20	28	0.30
Wound, diseases of the skin & subcutaneous tissue	14	19	0.28
Diseases of the genital system, pregnancy & childbirth	23	31	0.27
Diseases of the newborn & infant	15	20	0.26
Diseases of the digestive system	14	17	0.19
Impotence & sexual asthenia	8	9	0.13
Diseases of the musculoskeletal system	11	12	0.09
Diseases of the eye, ear & mouth	12	12	0.00
Diseases of the respiratory system	5	5	0.00
Mental & behavioural disorders	1	1	-

ICF: informant consensus factor.

The question of the availability of the resources was discussed with our experts. According to these specialists, excluded the exotic or domesticated plants (14.7%), 4 types for the 85.3% of native medicinal plants can be distinguished.

**Figure 4:**
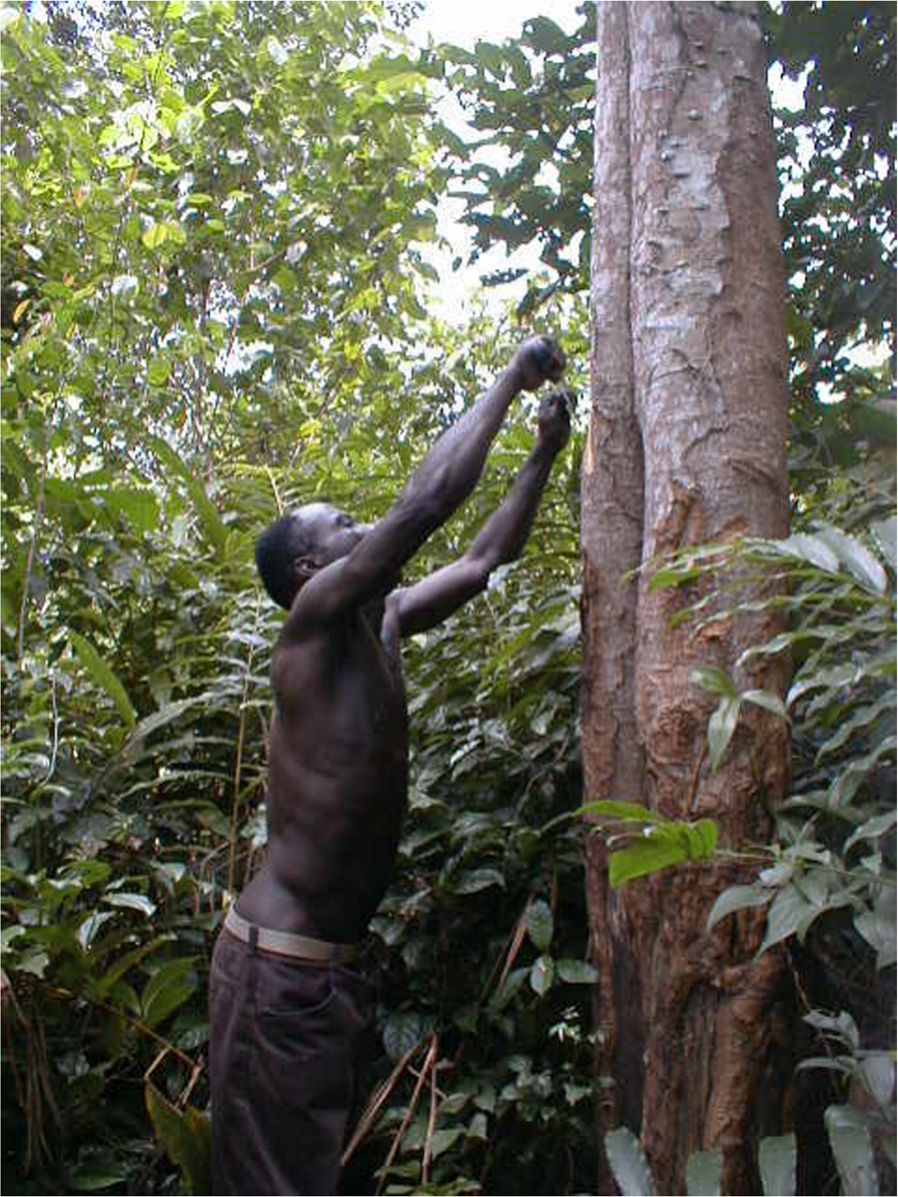
**Harvesting stem bark of** ***zanthoxylum gilletii*** **one the ehotile major medicinal plants.** This tree is becoming scarce in ehotile land and the stem bark difficult to harvest. Note the numerous torn stems at the base of the tree, due to frequent harvest.
- Endangered plants in the region, rarely found in their natural habitat (5.8%) such as *Garcinia kola* Heckel, *Khaya ivorensis* A.Chev. or *Picralima nitida* (Stapf) T.Durand & H.Durand.

- Abundant plants, with organs sought-after easy to collect (44.6%), such as *Ageratum conyzoides* L., *Alchornea cordifolia* (Schumach. & Thonn.) Müll.Arg. or *Baphia nitida* Lodd.
- Abundant plants, but organs sought-after difficult to harvest or rare (10.2%) such as *Tetracera alnifolia* Willd. or *Kigelia africana* (Lam.) Benth.
- Scarce plants in the region (24.7%) such as *Alstonia boonei*, *Momordica charantia* L., *Zanthoxylum gilletii* (Figure 4).

According to this information, 59.3% of medicinal plants used were easily accessible (44.6% of native abundant species and 14.7 of exotic domesticated or cultivated species), while 5.8% were hardly ever found in the region. About the categories of diseases, nearly 80% of plants used in the treatment of wounds and skin infections were easy to find, while 15% of species used as sexual stimulants (*Garcinia kola* and *Khaya ivorensis*) were very rare in the study area.

Concerning the used parts (Table 3), in the category of “abundant plants, with organs sought-after easy to collect”, leaves constituted 53.6% of parts used, while the roots were highly sought (50%) in the category of “endangered plants in the region, rarely found in their natural habitat”.

**Table 3:** **Proportion of medicinal plants part used in relation to plants availability type**

**Availability type**	**Proportion (%) of parts used**					
	**Leaf**	**Bark**	**Root & tuber**	**Fruit & seed**	**Exudate**	**Whole plant**
Abundant plants & part used easy to collect	53.6	16.1	19.6	1.8	1.8	7.1
Abundant plants. but part used difficult to harvest or rare	20.0	40.0	13.3	13.3	6.7	6.7
Scarce plants in the region	19.0	61.9	14.3	4.8	0.0	0.0
Endangered plants in the region. rarely found in their natural habitat	0.0	25.0	50.0	25.0	0.0	0.0
Exotic domesticated or cultivated plants	77.8	14.8	7.4	0.0	0.0	0.0

## Discussion

Most of ethnobotanical studies in West Africa are clearly marked as concerning the use of plants as medicine or as food [7]. Regarding medicinal uses, several works have been carried out about the treatment of several microbial infections [32,33], a range of tropical endemic diseases including malaria [34-38] as well as diabetes [39,40] and diverse infections [41].

This propensity of African ethnobotanists to medicinal plants reflects the fact that the study of plants used traditionally as medicines is an interesting discipline because of the possibility to find new drugs [42]. This has prompted many research teams to carry out studies on plants used in Africa by traditional healers against diseases.

Furthermore, these studies have the laudable aim of creating databases on traditional knowledge of plants use, although they remain disparate. Nevertheless, this abundant literature shows that despite the advances in Western medicine, African traditional medicine has gained renewed interest in the health care services throughout the continent. This could probably be due to the rapidly increasing awareness of the potential and curative abilities of alternative medicines, especially from the use of medicinal plants [42], as well as the inadequate access to Western medicine and physicians and the high cost for Western drugs [3].

This is the situation around the Aby Lagoon. Indeed, the results of this study showed that Ehotile people use plants in traditional medicinal practices. Globally, they know and use the handy plants as first treatment of common ailments. However, they also often resort to healer’s services. These healers are more or less known for specific field including reproductive health, rheumatism, and mental health. This specialization can justify the low values of ICF (<0.5). Indications of some plants, such *as Harungana madagascariensis*, *Ocimum gratissimum*, are widely shared, but information on plants and practices related to diseases specifically treated by healers is well protected. Thus, the recourse to these specialists is generally based on their area of recognized competence.

Though, as noted, the main purpose of these recourses remains what we called “African-disorders”: *Asema*, *elokposan* or *butumanklan. Asema*, for example, is a child disease manifested by an under nutrition. This condition would be due to “impurities” related to the resumption of sexual activity of the mother before her child is strengthened. This fact elicits a physical degradation of the child followed by frequent diarrhoea and signs of emotional deprivation. This disease is well known in sub-Saharan Africa. Such signs, related to the same cause, have been described in the Baka pygmies in Cameroon [43] and the Democratic Republic of Congo [44]. So these authors classified them as “African diseases” maintaining the local name (*Buse* in DRC) or assigning an approximate equivalence (“child-cross” in Cameroon).

According to the description of the signs, the *elokposan* would be the Pott’s disease that is a bone location of tuberculosis inducing developments of a hump. However, according to popular belief, *elokposan* is an occult disease. Thus, to protect themselves against it, wooden statues, also called *elokposan*, representing hunchbacks were suspended under the roofs on the side of the street.

*Butumanklan*, on the other hand literally means “anal wounds”. It would be the relaxation of the muscles of the rectum. A patient suffering from this illness struggles to contain the bowel gas.

Plants indicated for the treatment of these diseases are certainly well known, but malaria and its associated symptoms (fever, convulsion, jaundice) were most cited. In fact, 21 species (18% of all medicinal plants) are known for their use in the treatment of malaria. Among these species six are known only for this single indication: *Alstonia boonei*, *Azadirachta indica* A.Juss., *Bambusa vulgaris* Schard, *Harungana madagascariensis*, *Spathodea campanulata* P.Beauv. and *Vernonia conferta*. These species, which recorded a low use-value (UV = 0.02) are in fact highly specialized in the treatment of a single disease. This poses the problem of interpretation of use-value in the case of studies in medicinal plants. If it is true that a high use-value of a plant indicates its importance within a community [22,30], nevertheless, a UV close to 0 could indicate a high degree of specialization for a plant rather than a low use, when the frequency of citation of this plant is important. That is the case of *Harungana madagascariensis* which is widely known by Ehotile people as the anti-malarial plant.

This plant is used by other people of Côte d'Ivoire and Africa for the same indications, certainly because of its bright orange yellow latex (doctrine of signatures). It is used in the composition of many mixtures against jaundice in Côte d’Ivoire [11], Cameroon [43], southern Nigeria and Congo [45]. The stem bark contains several compounds such as anthracene, kenganone and kengathranol, anthronoïdes (harunmadagascarin A and B) having an important antioxidant activity [46]. Pharmacological works have confirmed the important traditional use of this plant in the treatment of malaria. Indeed, the stem bark extract exhibited significant anti-protozoan effects against *Trichomonas* and *Plasmodium* both in vivo and in vitro [47] and also exhibit anti-anaemic activity, and it’s known that anaemia is a universal feature of malaria [48].

In contrast to this species highly specialized, some plants recorded higher use-values (UV > 0.1). These plants included *Zanthoxylum gilletii* (UV = 0.15; 8 indications), *Ocimum gratissimum* (UV = 0.13; 7 indications), *Eclipta prostrata* (UV = 0.11; 6 indications) and *Paullinia pinnata* L. (UV = 0.2, 6 indications). Considered as panaceas, these species were variously used by the Ehotile. For example, *Ocimum gratissimum* is used for first intension in the treatment of several diseases (Convulsion, dysmenorrhoea, infant diarrhoea, gastritis, headaches). This plant is widely used in Côte d’Ivoire [12] and elsewhere in sub-Saharan Africa. For example, 7 indications for this plant are known in Ghana [49] and 11 indications in the south-eastern Nigeria [3]. *Zanthoxylum gilletii* is also widely known in Côte d’Ivoire for the diversity of its therapeutic indications [11,35]. The use of *Paullinia pinnata* in the treatment of malaria, haemorrhoids and sexual asthenia is not specific to Ehotile. In Benin, the plant is used to treat malaria and haemorrhoids, while in Mali and Togo, it is used against sexual asthenia. It is known now that antioxidant activity of the phenolic compounds contained in roots and leaves of this plant is the basis of its efficiency in the treatment of erectile dysfunction [50].

Other plants used by Ehotile are also known elsewhere or for similar purposes or for other pathologies. We will mention the example of *Icacina mannii*, *Cassia occidentalis* L., *Baphia nitida*, *Phyllanthus amarus* Schumach. & Thonn., *Picralima nitida* and *Momordica charantia*.

The use of *Icacina mannii* against sexual asthenia seems not widespread. However, its use in the treatment of dysmenorrhoea and infertility in women (two conditions linked according to local experts) was raised in West Africa [51].

The leaves of *Cassia occidentalis* are used in the treatment of malaria in Ghana [37] and Nigeria [36], as used by Ehotile and phytochemical studies have shown the anti-malarial properties of this plant [52]. As used by Ehotile, the leaf juice of *Baphia nitida* is applied against parasitic skin diseases [53], but the use of this plant to cure lumbar pains was not found elsewhere. *Phyllanthus amarus* is widely used as a medicinal plant and as known by Ehotile. This plant is taken to facilitate childbirth and it is considered as a good tonic, diuretic and antipyretic [54].

Therapeutic Indications of *Picralima nitida*, widely known by Ehotile in the treatment of inguinal hernia, are reported in Côte d’Ivoire [11]. The akuammine, alkaloid abundant in the seeds can explain analgesic and relaxant action of this plant. Indeed, pharmacological studies revealed that the extract or isolated compounds from this species have analgesic, anti-inflammatory, hypoglyceamic, hypotensive, antiplasmodial, antimicrobial, antiulcer and antitumorigenic activities [55]. The anti-malarial use of *Momordica charantia* is widely known. Furthermore, in vitro anti-plasmodial activity has been demonstrated [56].

Finally, most of plants used as medicine by Ehotile are known by other people of Côte d’Ivoire and Africa. Indeed, 72 i.e. 62% of medicinal species identified are listed in the 304 highly pharmaceutical plants of Côte d’Ivoire [12]. However, some uses in traditional medicines of certain plants, as well as some therapeutic indications seem exclusive to Ehotile culture.

For example, the roots of *Uvaria afzelii* are employed in all medicinal preparations against hydrocephalus. Yet, this indication has not been reported and moreover, this disease is very rarely mentioned in the literature. Similarly, no mention of the use of *Xylopia acutiflora*’s roots was found out. Nevertheless, these roots are widely known by Ehotile as sexual stimulants or as chewing sticks. *Xylopia acutiflora* is very known and appreciated by Ehotile women as chewing sticks as well for the pleasant minty sensation provided and the flexibility of the fibers obtained after chewing.

One of the issues raised in this survey is the availability of medicinal resources. As highlighted by various authors [3,4], access to medicinal resources is an acute problem in Africa. Loss of natural vegetation, due to various causes, is also a major factor in the erosion of traditional knowledge [57]. Indeed, it is always considered that the resources of the deep forest are more effective than ruderal or introduced species. For example, *Tetracera alnifolia* abounds, but mature plants containing the sought exudate are only found in old marshland, that are scarce and difficult to access.

Yet, despite the deterioration of natural habitats, the use of medicinal plants is still alive among Ehotile. This situation of seemingly low-impact of environmental degradation on medicinal practices was also observed in Zambia [14]. However, we believe that this may be largely due to the fact that the plants commonly used are often on hand or in fallow or neighboring plantations.

Conversely, exploitation for medicinal purposes of certain plant species led to their scarcity or their disappearance [57]. The plant parts used and the way in which medicinal products are harvested also affect availability of the resources. Medicinal products, including barks, roots, and exudates, are widely used, but little is known about the sustainability of harvesting strategies currently employed [57]. Particularly vulnerable are those species occurring at low densities, those whose roots are harvested [2,57]. In our case, more than 50% of the dwindling species were sought for their roots, which should increase the pressure on these species.

Also vulnerable are those species whose bark or oil is extracted unsustainably [2] *Harungana madagascariensis*, *Zanthoxylum gilletii* and *Icacina mannii* are an illustration of unsustainable extraction, in our case. *Harungana madagascariensis*, as mentioned, is widely used as an anti-malarial, by specialized healers and housewives. Unfortunately, the stem bark, is easily removed and wholly extracted as well as leaves, leading to loss of the tree. According to the healers, currently, the plant only exists in the vicinity of a few marshy formations. *Zanthoxylum gilletii* is similar case but this species owes its survival to its resilience and its height. *Icacina mannii* is prized for its tuber, deemed effective against haemorrhoids and impotence. The collection of the whole tuber prevents any possibility of regeneration.

However, as noted elsewhere [57], the ecology of even the most widely used species is poorly understood and the impact of medicinal plant harvesting practices on the availability of resources are not known as well. Nevertheless, the relative impacts of harvesting medicinal plants are insignificant when compared with the impacts of deforestation [57].

## Conclusions

The study of medicinal plants in Côte d’Ivoire covers many areas ranging from simple monographs to phytochemical and pharmacological studies. Few works have tried to estimate the knowledge about plants by a quantitative approach. This way of doing ethnobotany is in its early days in sub-Saharan Africa and should experience strong changes like what is done in Europe, Brazil and North America for example.

The study of the use of medicinal plants in Ehotile was interesting given their lifestyle (fishing) and especially the degradation of their environment.

The disappearance of natural formations in Ehotile land could indicate a low knowledge and use of medicinal plants by Ehotile. Rather, medicinal plants play an important role in the daily life of Ehotile thereby coming as complementary and alternative to modern health system. 123 plants were inventoried. They are used, in addition to medical prescriptions or exclusively, for treating several ailments most common of which are; malaria, sexual asthenia, hydrocephalus, pregnancy disorders and dysmenorrhea. Some of these plants are known for only one indication whereas, others have multiple usages.

The information was collected from two informants groups: the laypeople and key informants. Key informants helped us to confirm much of the information received from laypeople. The Informant Consensus Factor was only applied to this group and it was confirmed that they were, each highly specialized in the treatment of various diseases. With these local experts, the availability of identified plants was also defined. Some medicinal plants were easily accessible, whereas, others were scarce or no longer found in the region. It is feared that the scarcity of plants is a precursor to the disappearance of the rich knowledge and practices associated with them.

Two other aspects of this study are ongoing and should allow, with key informants, to specify the extent of the impact of environmental degradation on Ehotile medicinal practices firstly, and secondly to estimate the effect of medicinal plants harvesting on the availability of the most used species.
